# Kangaroo Mother Care Induced Serum Oxytocin Facilitates Prolactin and IL-10 Among Emergency Cesarean Mothers [Letter]

**DOI:** 10.2147/JMDH.S483821

**Published:** 2024-07-01

**Authors:** Norlaila Sofia

**Affiliations:** 1 Midwifery Department, Poltekkes Kemenkes, Banjarmasin, Indonesia

## Dear editor

I have carefully reviewed the article entitled “Kangaroo Mother Care Induced Serum Oxytocin Facilitates Prolactin and IL-10 Among Emergency Cesarean Mothers” by Ramaiah et al.^1^ While the study provides valuable insights into the hormonal and cytokine changes associated with Kangaroo Mother Care (KMC) practice in emergency cesarean section mothers, there are several limitations that should be addressed.

Firstly, the study lacks a clear description of the inclusion and exclusion criteria for the control group. It is essential to ensure that the control group is comparable to the KMC group in terms of maternal age, gestational age, and other relevant factors that may influence the hormonal and cytokine levels.^2^ Additionally, the small sample size (16 in the KMC group and 9 in the control group) raises concerns about the statistical power and generalizability of the findings.

Secondly, while the authors acknowledge the limitation of obtaining serum samples at relatively long intervals, this could have affected the accuracy of the measurements, especially for short-lived hormones like oxytocin. More frequent sampling would have provided a better understanding of the kinetics of hormonal and cytokine changes.^3^

Thirdly, the authors did not provide information on the potential confounding factors that could influence the hormonal and cytokine levels, such as maternal stress, pain, and medications used during the perioperative period.^4^ These factors should be carefully controlled or accounted for in the analysis to ensure the validity of the results.

Fourthly, the study lacks a detailed description of the KMC protocol used, including the duration, frequency, and timing of the sessions. Standardization of the KMC protocol is crucial for replicability and comparison with other studies.^5^

Finally, the authors did not assess the long-term effects of KMC on maternal and infant outcomes, such as breastfeeding duration, maternal mental health, and infant development.^6^ Longitudinal studies are needed to understand the sustained benefits of KMC and its impact on various aspects of maternal and infant well-being.

Despite these limitations, the study provides valuable preliminary data on the potential benefits of KMC in emergency cesarean section mothers. Future well-designed, prospective studies with larger sample sizes, controlled confounding factors, and standardized KMC protocols are needed to substantiate the findings and further elucidate the mechanisms underlying the observed hormonal and cytokine changes.
